# Physiotherapy as an Effective Method to Support the Treatment of Male Urinary Incontinence: A Systematic Review

**DOI:** 10.3390/jcm12072536

**Published:** 2023-03-27

**Authors:** Agnieszka Mazur-Bialy, Sabina Tim, Daria Kołomańska-Bogucka, Bartłomiej Burzyński, Tomasz Jurys, Natalia Pławiak

**Affiliations:** 1 Department of Biomechanics and Kinesiology, Faculty of Health Science, Jagiellonian University Medical College, Skawińska 8, 31-066 Krakow, Poland; 2 Department of Rehabilitation, Faculty of Health Sciences in Katowice, Medical University of Silesia, 40-055 Katowice, Poland; 3 University Hospital in Krakow, Jakubowskiego 2, 30-688 Krakow, Poland

**Keywords:** urinary incontinence, men, physiotherapy, prostatectomy, pelvic floor muscle training, biofeedback, electrostimulation

## Abstract

Urinary incontinence (UI) is a serious health issue that affects both women and men. The risk of UI increases in men with age and after treatment for prostate cancer and affects up to 32% of men. Furthermore, UI may affect up to 69% of men after prostatectomy. Considering such a high incidence, it is critical to search for effective methods to mitigate this issue. Hence, the present review aims to provide an overview of physiotherapeutic methods and evaluate their effectiveness in treating UI in men. This systematic review was performed using articles included in PubMed, Embase, WoS, and PEDro databases. A total of 6965 relevant articles were found. However, after a risk of bias assessment, 39 studies met the inclusion criteria and were included in the review. The research showed that the available physiotherapeutic methods for treating men with UI, including those after prostatectomy, involve pelvic floor muscle training (PFMT) alone or in combination with biofeedback (BF) and/or electrostimulation (ES), vibrations, and traditional activity. In conclusion, PFMT is the gold standard of UI therapy, but it may be complemented by other techniques to provide a personalized treatment plan for patients. The effectiveness of the physiotherapeutic methods varies from study to study, and large methodological differences make it difficult to accurately compare individual results and draw unequivocal conclusions.

## 1. Introduction

Urinary incontinence (UI) affects up to 32% of men, although it presents more often in women [1,2]. A significant ailment affecting up to 69% of men is post-prostatectomy incontinence (PPI), following radical prostatectomy (RP), prostate irradiation, and surgery for benign prostatic hyperplasia [3]. However, as there is no precise definition of continence/incontinence, the number of men with UI may vary [4]. Prostate cancer is the most commonly diagnosed cancer in men [5]. RP generally bodes well in terms of oncological outcomes, but patients are still at risk of surgical and postoperative complications [6]. RP can damage the nerves and structures around the pelvic floor, leading to a weakened urethral sphincter and urethral bulb [7]. On the other hand, radiation therapy damages DNA, causing inflammation and structural changes in collagen [3]. UI affects quality of life and can intensify depression and anxiety [8]. Considering that pelvic floor physiotherapy is much less researched in men than in women, it is necessary to create guidelines and summarize possible physiotherapeutic solutions for the treatment of UI in men. The UI European Society of Urology (EAU) and American Association of Urology (AUA) guidelines recommend pelvic floor muscle therapy (PFMT) as first line treatment after prostatectomy [9]. These recommendations are very similar to the treatment of UI in women [10]. However, the anatomical differences of the pelvic floor between genders have to be taken into account. Most articles describing the main principles of PFMT are based on recommendations assuming female anatomy, which is undoubtedly a limitation for treating UI in men [11]. PFMT should be performed pre-operatively and immediately after catheter removal to optimize its effectiveness [12]. PFMT can be combined with biofeedback or electrostimulation. However, there are no specific recommendations as to the pelvic floor muscle training instructions, the number of repetitions, and optimal positions for exercises [13].

For this reason, the present study aims to review and identify physiotherapeutic methods in the treatment of men with UI, including those following prostatectomy, and to evaluate their effectiveness.

## 2. Materials and Methods

The review protocol was based on the Preferred Reporting Items for Systematic Review and Meta-Analysis (PRISMA) [14] and registered in PROSPERO (protocol no. CRD42021278370). Inclusion criteria were based on the participant–intervention–comparator–outcomes–study design (PICOS) format.

Participants: Only the following were considered: studies including men over 18 years of age, studies including men with UI or post-prostatectomy UI, and studies including both men and women, but where the results were separated by gender. Studies with men younger than 18 years of age or involving animal or in vitro models were excluded.

Intervention: Studies containing any physiotherapeutic intervention were included. Studies without intervention or where it was combined with pharmacotherapy and data analysis was not able to separate outcomes were also excluded.

Comparison: No intervention, relative to other methods, conservative treatment, and placebo.

Outcomes: Assessment of therapy effectiveness, UI symptoms, QoL, subjective assessment of health, urodynamic parameters, and quality of pelvic floor muscle contraction.

Study design: Randomized controlled trials, published in English or Polish. Non-experimental studies and reviews were excluded.

The search was performed on 4 databases: PubMed, Embase, Web of Science, and PEDro. Articles in English and Polish up to 2022 were considered, and the lower limit of the publication date was not specified. The search was concluded on the fourth week of October 2022. Results from keyword searches were exported into Excel. Duplicates were then removed. Keywords were: (men) AND (urinary incontinence OR prostatectomy) AND (physiotherapy OR electrotherapy OR biofeedback OR pelvic floor muscle training OR magnetic stimulation OR conservative treatment OR physical therapy OR vibration OR rehabilitation).

Inclusion criteria were as follows: randomized controlled trials, studies involving adult men with UI, studies assessing the symptoms of PPI, studies assessing the effectiveness of physiotherapy, and manuscripts in English or Polish. Exclusion criteria included: studies other than RCTs, studies including women and where it was not possible to extract data by gender, studies without physical therapy, studies where QoL or UI symptoms were not measured, studies on people younger than 18 years of age, and studies in a language other than English or Polish.

The search was conducted independently by three researchers. The first screening of articles was evaluated by titles and abstracts against inclusion and exclusion criteria. After screening, full text articles were retrieved. Further inclusion depended on the type of study, detailed information about the participants, interventions, methods of evaluation and questionnaires used, and description of results and main conclusions. Finally, only RCTs were included, which were then assessed for the potential risk of bias. Any doubts and inconsistencies between the researchers were resolved by discussion or the inclusion of a fourth investigator.

Two researchers independently performed risk of bias analysis using the Risk of Bias 2 tool (Cochrane platform). Researchers evaluated 5 domains: randomization process, deviations from intended interventions, missing outcome data, outcome measurement, and selection of the reported result. Each domain consisted of questions that could be answered as follows: Yes/Probably Yes/Probably No/No/No Information. Based on the responses, each domain was rated as “low”, “high”, or “some concerns” regarding bias risk. All domains had to be rated as low risk for the study to be considered at a low risk of bias [15]. After the analysis was completed, researchers compared their answers. In cases of conflicting results, they either reached a solution or consulted with a third researcher. A detailed description of the risk of bias assessment can be found in Appendix A.

## 3. Results

**Figure 1:**
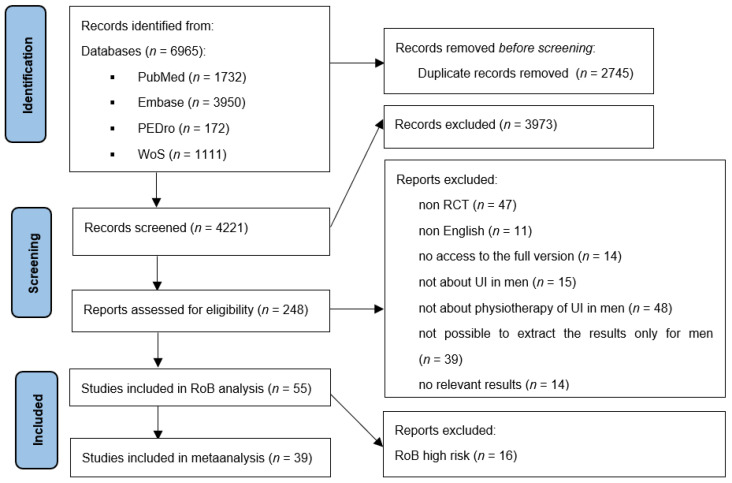
PRISMA flow diagram of selection study.

The search identified 6965 records. After removing duplicates, 4220 studies remained. A total of 3973 studies were rejected after reviewing the titles and abstracts. A total of 247 papers remained to be fully read. After applying the inclusion and exclusion criteria, 193 works were disqualified. The remaining 55 papers were subjected to a risk of bias analysis. Based on the results of the RoB-2 risk of bias assessment tool, 33 studies were labeled as low risk, 6 as being of some concern, and 16 as high risk. Articles with a high risk of RoB were rejected. To increase the number of works describing physiotherapy in PPI, we decided to include works that were labelled with “some concerns” in the RoB-2 analysis. A total of 38 studies ultimately met all criteria. A detailed analysis of the review is presented in the PRISMA diagram (Figure 1). Table 1 presents a brief description of the relevant studies. The RoB-2 risk of bias in every domain and the total percentage plot of the overall risk of bias are given in Appendix A.

### 3.1. Pelvic Floor Muscle Training in Men with UI

PFMT is the most basic, noninvasive conservative treatment of PPI [55]. The aim of pelvic floor muscle training (PFMT) in PPI is to improve continence by focusing on how PFM are involved in motion [11] as well as increasing strength, endurance, and the autonomic coordination of pelvic floor muscles and urethral sphincters [56]. PFMT contributes to better compression and an increase in pressure inside the urethra when there is a sudden increase in intra-abdominal pressure [57]. This highlights the need to integrate PFMT into daily activities that cause increased intra-abdominal pressure [11]. Standard PFMT usually consists of PFM exercises and the palpation of contractions with or without biofeedback (BF) [58]. However, the number of repetitions and their duration should be tailored to the patient. In order for PFMT to be effective, PFM exercises should be performed several times a day for several months [59]. In many studies, men were asked to focus on contracting in the anal sphincter area as if treating fecal incontinence [13], while PFMT after prostatectomy should focus on the urethra [11].

While PFMT performed before a prostatectomy can reduce the symptoms of UI after surgery, it is critical to continue therapy postoperatively [21,26]. Milios et al. [35] observed that the effectiveness of preoperative PFMT is influenced by the intensity of the exercises. Patients who participated in training sessions before the procedure and performed quick PFM contractions reported better continence improvement and QoL than men who underwent less intensive training [35]. Postoperative therapy should be started as soon as possible after removing the catheter [18,19,26,35] and continued until UI is resolved [19]. Perioperative physiotherapy effectively reduces problems with the urinary system compared to standard care [26]. The American Urological Association (AUA) recommends implementing PFMT before and 3 to 4 weeks after prostatectomy [60]. Regularly performing PFMT after prostatectomy reduces the need to use pads or other hygienic materials, which improves QoL and reduces social isolation [19]. Preoperative PFMT that was continued after surgery, compared to postoperative physiotherapy alone, is also better at minimizing UI in men with benign prostatic hyperplasia [18].

Most authors recommended that supervised training should be performed independently at home daily [19,20,21,23,26,29,32,37,42,46] and that supervised training is much more effective and long-lasting than unsupervised PFMT. In most studies, home PFMT consisted of three sets of 10 [37], 15 [43], or 20 PFM contractions in a single session [19]. Moore et al. [36] found that verbal and written instructions on PFMT and home exercises, combined with telephone call by nurse, is as beneficial as physiotherapy [36]. Heydenreich et al. [28] noted that unsupervised PFMT also improves UI; however, better outcomes were achieved with a physiotherapist session [28]. Overgard et al. [39] concluded that regular unsupervised PFMT reduces UI symptoms, but long-term effects were maintained when patients underwent physiotherapist-guided PFMT [39]. Apart from PFMT, physiotherapists can use supervised Pilates training [42] to diversify the exercises with appropriate accessories, e.g., an oscillating rod. Combining deep abdominal muscle and PFM activation increased the effectiveness of PFMT and reduced UI symptoms [28]. PFMT could be combined also with acupuncture [20]. A detailed description of the analyzed studies is presented in Table 2.

### 3.2. Pelvic Floor Muscle Training and Biofeedback

A frequent early problem that limits the effectiveness of pelvic floor muscle exercises is the inability to properly contract and relax the correct muscle groups. Biofeedback is often utilized to help teach proper PFM activation [55]. The equipment used in BF provides visual and auditory feedback on PFM functionality, which is subsequently used to develop training parameters [61]. Visual information about PFM activity during exercise allows patients to correctly contract, increase body awareness, and motivate themselves to continue PFMT [55]. Verbal feedback from a therapist during the palpation of the levator ani muscle was also notably mentioned [24]. Floratos et al. [24] showed that the combination of PFMT with electromyography (EMG) and verbal feedback may have similar effectiveness in minimizing the symptoms of PPI [24]. Moore et al. [36] noted similar outcomes between physiotherapist-supervised PFMT, including BF and exercises performed at home following written instructions and supervised by a nurse via telemedicine [36].

Evaluating the effectiveness of PFMT combined with BF in the treatment of PPI was the aim of many articles [17,22,24,25,36,38,41,44,45,51]. Most authors conducted training with BF once a week [25,36,44,45,51], which lasted between 20 [51] and 30 min [17,24,25,44,45]. PFMT with BF is used in patients both before [17,22,41] and after radical prostatectomy [17,25,45,51]. Perez et al. [41] noted that preoperative PFMT with BF may be effective in reducing the symptoms of UI and erectile dysfunction as compared to no therapy after prostate removal [41]. However, there are studies that did not confirm these data [22]. Studies also differed in the number of sessions. This allowed us to note features such as two sessions of PFMT with BF [22] when compared to ten [41], which did not reduce the risk of developing UI. To improve urinary continence in men after prostatectomy, PFMT with BF should be continued after surgery [25,51]. Tienforti et al. [51] observed that a single preoperative PFMT session with BF and monthly postoperative sessions combined with home exercises effectively reduce the symptoms of PPI as compared to standard care and unsupervised exercise [51]. Oh et al. [38] noted that the BF device reduced UI symptoms compared to only verbal and written instructions, with differences being mainly visible after the first month of treatment [38]. However, this study was flagged as having some methodological concerns. In turn, Ribeiro et al. [45] showed that conducting early rehabilitation (PFTM with BF, 2 weeks after surgery) significantly improved continence, voiding symptoms, and pelvic floor muscle strength 12 months after surgery [45].

BF is a means of increasing a patient’s awareness of their PFM, thus making PFMT more effective. However, if the patient remains unable to perform an isolated pelvic floor contraction, other techniques should be chosen to activate and restore PFM functionality. A detailed description of these studies is presented in Table 3.

### 3.3. Electrical Stimulation in Men with UI

The electrical stimulation (ES) of the pudendal nerve triggers the maximal contraction of the PFM, improves urethral pressure, and reduces detrusor overactivity [62]. Electrotherapy leads to passive PFM contraction, which is significant for patients with difficulties consciously contracting and relaxing their PFM [63]. Therefore, it is often used in UI therapy [62]. Electrical stimulation in male pelvic floor disorders is performed using surface electrodes, rectal electrodes, and transcutaneous electrical nerve stimulation. In men suffering from UI, electrostimulation is applied at the level of the spinal cord or nerves that control the lower urinary tract. The indirect electrotherapy of the pudendal nerve is believed to activate the PFM to then contract the urethra [64]. ES is, however, contraindicated when there is an oncological concern, as it may stimulate malignant cell proliferation [64].

In most of the analyzed research protocols, electrostimulation was combined with other PFM therapeutic techniques [16,27,30,33,40,48,52] and lasted between 15 [16,31,40,52] and 20 min [27,33,34]. Ahmed et al. [16] observed that UI therapy outcomes may improve after combining ES therapy with BF [16]. Mariotti et al. [33] confirmed these findings in the early therapy of men after prostate surgery, with significant continence improvements appearing between the 4 week and 6 month follow-ups [33].

ES is most often combined with PFMT in the treatment of UI [27,30,31,40,48]. It has been shown that transcutaneous and anal electrostimulation when combined with PMFT significantly reduces UI symptoms in men after RP [40]. Soto-Gonzales et al. [48] observed that the combination of BF with ES and PFMT home instruction significantly reduces UI symptoms and improves patients’ quality of life. ES with PFMT also reduces erectile dysfunction [30]. PFMT effectiveness when combined with active and sham ES was also compared. Despite the observation that UI symptoms were lesser in men who received active ES months at 6 months, no similar differences were obtained after 12 months [52]. Laurienzo et al. [30] noted that regardless of whether patients received PFMT only at home or if PFMT included ES, continence improved as early as 3 months postoperatively [30].

It should be noted that the parameters of ES differ depending on the type of UI. In men with SUI, the frequency of 50 Hz is used; in patients with UUI, 4 Hz; and in patients suffering from mixed UI, therapy with both the above parameters can be used [27]. A detailed description of the analyzed studies is presented in Table 4.

## 4. Discussion

The purpose of this review was to determine what physiotherapeutic methods are used to treat PPI and assess their effectiveness. PPI can occur in 69% of men [65]; however, the true number may be different as there is no standardized definition of incontinence [4]. The most commonly assessed physiotherapeutic method in our review was PFMT, followed by ES and BF. Each of these techniques can help to improve continence after surgery.

Physiotherapy for men with prostate cancer should be performed before and after prostatectomy. PFMT before and after surgery is also important for patients with benign prostatic hyperplasia [18]. Studies showed the benefit of preoperative PFMT [21,35] to prepare the pelvic floor for postoperative physiotherapy. Centemero et al. [21] compared the effectiveness of pre- and postoperative training with only postoperative exercises. It has been shown that starting PFMT 30 days before the planned surgery and continuing it postoperative significantly minimizes the risk of UI and improves the QoL in men as compared to postoperative physiotherapy alone [21]. Similar outcomes were described by Milios et al. [35]. However, the study by Anan et al. [18] also showed that PFMT 28 days before and after the HoLEP procedure for BPH, compared to postoperative physiotherapy alone, minimizes the risk of UI, particularly at 3 months after the procedure.

Preoperative exercises increase neuromuscular reserves, teach the correct pattern of PFM contraction, increase muscle mass, and improve blood circulation in the pelvic floor area [55]. Despite the many benefits of preoperative PFMT, it only has a conditional level C recommendation (AUA) [60]. Preoperative PFMT can be combined with BF [22,25,41,51] and ES [30,52]. Preoperative PFMT combined with BF reduces the severity of UI after surgery and shortens the time needed until patients are able to control voiding [21]. Gezginci et al. [26] also noted that preoperative training, compared to standard care, improves both the continence and QoL of patients after RP.

The effectiveness of pre- and postoperative BF was also compared to postoperative BF alone [25]. One study found that combined pre- and postoperative training and postoperative training alone were similarly effective in minimizing UI symptoms [25]. The beneficial outcomes of preoperative BF compared to no physiotherapy were achieved after 10 training sessions consisting of maximal, fast, and slow PFM contractions [41]. However, similar results were not found by de Lira et al. [22]. The control group did not receive any physiotherapy, while only two supervised PFMT combined with BF were introduced before the surgery in the experimental group. PFMT was continued at home both before and after surgery [22]. Yamanishi et al. [52] combined preoperative PFMT with active and sham ES. Six months after surgery, significantly fewer UI symptoms were observed in men who received active ES. No similar differences were observed after 12 months. Men from both groups performed identical PFMT before and after surgery [52]. BF is more effective in reducing PPI symptoms than no treatment, while its preoperative efficacy should be more carefully investigated.

We, like numerous other studies, have observed the effectiveness of PFMT [6]. Verbal and written instructions, feedback about contractions, and performing PFM exercises at home (three series of fifteen repetitions) increase PFM strength, reduce UI symptoms, and improve QoL in men with prostate cancer after surgery as compared to no post-treatment physiotherapy [43]. A similar result was obtained by Manassero et al. [32]. Moreover, they showed that the benefits of regular PFMT in men persist one year after RP [32]. Faithfull et al. [23] observed a significantly greater improvement in UI symptoms after 3 months of physical therapy compared to standard care. Unfortunately, no similar differences were found after 6 months [23]. Three sets of exercises each lasting at least 3 min per day were also beneficial in the treatment of UI due to BPH [18]. PFMT has only a moderate evidence level B recommendation [60]. PFMT is recommended as a treatment for PPI in men according to the European Association of Urology, but there are no specific recommendations as to how it should be performed [66]. PFMT supervised by a physiotherapist may have better results in improving continence than training alone [37]. More research is required to determine the most effective PFM exercise plan [6].

PFMT is often combined with BF and ES. Van Kampen et al. [29] implemented anal ES in addition to PFMT in patients who could not activate PFM correctly or whose PFM strength was too low [29]. Bernardes et al. [20] combined PFMT with acupuncture in the treatment of UI after RP, noting that while PFMT alone is beneficial, patients who also used acupuncture had better results after 8 weeks. Pedriali et al. [42] showed that similar therapeutic effects after RP could be obtained both through Pilates training and standard PFMT in combination with ES [42]. Gomes et al. [27] noted that Pilates training or PFMT with ES significantly improved continence as compared to no postoperative rehabilitation [27]. If used in combination with PFMT, both transcutaneous perineal and anal electrostimulation have similar efficacy in the treatment of UI in men after RP [40]. The type of UI should first be determined to correctly select the therapy parameters [27,42]. In SUI, the most frequently used frequency is 50 Hz; in UUI, it is 4 Hz; and in MUI, both can be used [27,42]. ES can also be combined with BF [33]. The use of a 15-min BF followed by a 20-min ES beginning on the 7th day after decatheterization and continuing for 6 weeks eliminates the symptoms of UI in men after RP surgery. Combining ES with PFM can help patients who have difficulty in contracting PFM properly.

BF can also be combined with PFMT [16,19,21,35,41,46]. Studies have indicated that BF-assisted training can improve short-term and long-term continence more effectively than PFMT alone [67]. It has been observed that both verbal BF and combined EMG are beneficial after surgery [19]. The use of BF in the treatment of UI in men enables them to learn appropriate exercise patterns, improve motivation, improve QoL, and gain a sense of control in their therapy [68]. Nevertheless, the American Urological Association does not recommend adding BF to training [60].

Bladder training and supervised PFMT lasting at least 3 months is recommended as first-line therapy for people with UI. However, studies have shown that some of the therapies do not produce the desired effects or that results obtained with the same methods are contradictory [69].

## 5. Limitations of the Study

The current review has some limitations. The main one is the inclusion of only RCT studies, which reduced the total number of analyzed publications. Articles where it was impossible to isolate the results only for men were also rejected. We also wanted to compare how physiotherapy influenced UI treatment in men with prostate cancer. We therefore did not set any time limits for publication. Unfortunately, despite attempts to contact the authors of several articles, it was not possible to obtain full access to all publications. This issue affected mainly old publications.

RoB analysis was also performed to include the highest quality of studies, leading us to reject 16 articles. Notably, when compared to the meta-analyzes by Wang et al. [70] and Zhu et al. [71], we did not limited our work to just one physiotherapeutic method. We presented papers in which the therapy for men with UI was PFMT, BF, ES, and acupuncture, as well as their various combinations. Unfortunately, a further significant limitation when comparing the results was the variability of treatment regimens, differences in the measurement of treatment effects and different durations for follow-up. Similar problems were also reported by Kannan et al. [64]. Another important limitation when comparing the results was the lack of a clear definition of urinary incontinence in men, which may even distort the estimation of the scale of this problem [4].

## 6. Conclusions

In conclusion, according to the present review, physiotherapy is an important element in the fight against UI, especially following prostatectomy. The authors of this review demonstrated that PFMT, PFMT plus BFB and ES, and PFMT plus ES are effective approaches for reducing UI. This is a key element for preoperative preparation and personalized postoperative management. As it mainly focuses on improving the PFM function and innervation, the type and intensity of training and/or supplemental techniques should be selected individually depending on the patient’s needs. Nevertheless, considering the small number of studies and inconsistent methodology behind assessing the effectiveness of therapy, it is necessary to conduct further invaluable research to optimize the physiotherapeutic approach for treating UI.

## Figures and Tables

**Table 1:** Characteristics of outcome measures of studies of physiotherapy in male urinary incontinence that qualified for this review.

Study	PT	TS	Qol	ED	GS	Other	RoB
Ahmed [16]	+	+	+			UE	Low
Allameh [17]	+				+		Some concerns
Anan [18]			+			ICIQ-SF, IPSS, UE, OABSS, G8 score	Some concerns
Aydin Sayilan [19]		+	+		+	TU	Low
Bernardes [20]	+					Daily Pad Used	Low
Centemero [21]	+		+		+	UE, MMSE, PGI-I, BD	Low
De Lira [22]		+	+	+	+		Low
Faithfull [23]			+			IPSS, ICSmaleSF, SESCI	Low
Floratos [24]	+					UE	Low
Geraerts [25]	+		+			IPSS, VAS	Low
Gezginci [26]			+			ICIQ-SF, ICIQ-MLUTS	Low
Gomes [27]	+	+	+			BD	Low
Heydenreich [28]	+	+	+			FACT-P	Low
Kampen [29]	+	+				VAS	Low
Laurienzo [30]		+	+	+		IPSS	Low
Laurienzo [31]	+	+	+		+		Low
Manassero [32]	+	+	+			VAS, IPSS	Low
Mariotti [33]	+	+			+		Low
Mariotti [34]	+	+			+	UE	Some concerns
Milios [35]	+		+		+	IPSS, EPIC-CP, BD	Low
Moore [36]	+		+		+	IPSS, BD	Low
Nilssen [37]		+	+	+	+	UCLA-PCI	Low
Oh [38]	+	+	+	+	+	IPSS	Some concerns
Overgard [39]	+	+			+	UCLA-PCI	Low
Pane-Alemany [40]	+		+			ICIQ-SF	Low
Perez [41]			+	+			Low
Pedriali [42]	+		+			BD	Low
Porru [43]			+			AUA, uroflowmetry, BD	Low
Rajkowska-Labon [44]	+					BD	Some concerns
Ribeiro [45]			+				Low
Santos [46]	+				+		Low
Serdà [47]	+	+	+			FACT-P, WP, 8RM, VAS-UI	Low
Soto González [48]	+		+			BD	Low
Strojek [49]		+	+		+	MC, EPIC-26, BDI II	Low
Tantawy [50]	+		+			I-VAS	Low
Tienforti [51]		+	+		+	UCLA-PCI, IPSS	Low
Yamanishi [52]	+		+				Low
Yokoyama [53]	+	+	+			BD	Low
Zhang [54]	+	+	+		+	SPSMQ, BD, UCLA-PCI, IPSS, VAS	Some concerns

AUA, the American Urological Association Symptom Score; BD, bladder diary; BDI II, Beck’s Depression Inventory; ED, erectile dysfunction; EPIC-CP, Expanded Prostate Cancer Index Composite for Clinical Practice; EPIC-26, Expanded Prostate Cancer Index Composite; FACT-P, Functional Assessment of Cancer Therapy-Prostate questionnaire; GS, Gleason Score; ICIQ-SF, International Consultation on Incontinence Questionnaire Short Form; ICIQ-MLUTS, International Consultation on Incontinence Questionnaire for Male Lower Urinary Tract Symptoms; ICSmaleSF, International Continence Society Male Short Form questionnaire; IPSS, International Prostate Symptom Score; I-VAS, Incontinence Visual Analogue Scale; MC, myostatin concentration; MMSE, Mini-Mental State Examination; OABSS, overactive bladder symptom score; PT, pad test; PGI-I, a Patient’s Global Impression of Improvement; RoB, risk of bias; SESCI, self-efficacy measured by the Self-Efficacy for Symptom Control Inventory; SPSMQ, Short Portable Mental Status Questionnaire; QoL, quality of life; TS, tumor stage; TU, transabdominal ultrasonography; UCLA-PCI, University of California, Los Angeles—Prostate Cancer Index; UE, urodynamic examination; VAS, visual analog scale; VAS-UI, visual analog scale of urinary incontinence; WP, waist perimeter; 8RM, muscular resistance.

**Table 2:** PFMT in men with UI.

Reference	Main Objective	Participants	Intervention	Follow-Up	Outcomes
Anan et al. (2020), Japan [18]	Assessment of the impact of preoperative PFMT on improving UI in men after HoLEP	70 men with BPH GrA: 35 (aged 72, range 62–83 yr) GrB: 35 (aged 73, range 57–86 yr)	GrA: PFMT—preoperatively (28 days), postoperatively (2nd day after HoLEP). GrB: PFMT—postoperatively (2nd day after HoLEP). In both groups—instructions of PFMT, 3 min of PFMT, at least 3× a day. Assessment: ICIQ-SF, urodynamic examination	3 days after removal of the catheter, 1st, 3rd, and 6th mth after HoLEP	Preoperative PFMT before HoLEP procedure results in a lower rate of urinary incontinence in men, especially 3 months after surgery.
Aydın Sayılan et al. (2018), Turkey [19]	Assessment of the impact of PFMT on the occurrence of UI in patients after RP	60 men after RP Con: 30 (aged 59.93 ± 6.98 yr) Exp: 30 (aged 63 ± 8.61 yr)	Con: no PFMT, breathing exercises, operation info. Exp: 1–4 PFMT sessions (1 h)—activate PMF in functional positions, 20 contractions of 10 s in 3 positions; 3× of PMFE at home 20× daily—6 mth. Assessment: IDQ, ICIQ-SF, PT	10 days after catheter removal, 1st, 3rd, and 6th mth AS	Regular PFMT performed for 6 months after RP surgery significantly minimizes UI problems in men.
Bernardes et al. (2022), Brazil [20]	Assessment of the effectiveness of acupuncture therapy and PFMT in the treatment of UI in men after RP	63 men after RP Con: 31 (aged 63.93 ± 7.23 yr) Exp: 32 (64.84 ± 6.4 yr)	Con: 8 PFMT sessions with physiotherapist, daily PFMT at home for 8 weeks. Exp: 8 PFMT sessions with physiotherapist, daily PFMT at home for 8 weeks, 8 sessions of acupuncture. Assessment: PT, daily PT, sociodemographic and clinical questionnaire	Before, 4th, and 8th weeks of therapy	Both PFMT and PFMT combined with acupuncture reduce UI in men after RP. However, after 4 and 8 weeks of therapy, a greater improvement was observed in patients using PFMT associated with acupuncture.
Centemero et al. (2010) Italy, [21]	Assessment of the benefits of PFMT in men after RP who started therapy before vs. those who started postoperatively	118 men after RP Con: 59 (aged 57.5, range 46–67 yr) Exp: 59 (aged 60.5, range 48–68 yr)	Con: sPFMT 2× a week for a month AS, daily PFMT at home. Exp: PFMT 30 days BS: sPFMT 2× a week for 30 min, 30 min of PFMT daily at home; AS: sPFMT 2× a week for a month, daily training at home. Assessment: MMSE, PE, urodynamic testing, 3 d bladder diary, ICS-male-SF, PGI-I, QoL, 24 h PT	1st and 3rd mth AS	Men who started PFMT BS had a lower risk of UI after RP. Early PFMT reduced UI problems compared to men who only exercised AS.
Faithfull et al. (2022), United States [23]	Evaluation of the effectiveness of rehabilitation in improving the symptoms of the urinary system in patients with prostate cancer	63 men with prostate cancer after radiotherapy Con: 32 (aged 72.2 ± 6.7 yr) Exp: 31 (aged 69.9 ± 7.3 yr)	Con: standard care—control visits to the hospital, telephone support. Exp: 4 group sessions (60–90 min), 1 individual session (40 min), 2 phone sessions for 10 weeks—education, PMEs, homework; then 4 months of self-therapy at home. Assessment: IPSS, ICS-male-SF, EORTC QLQ-PR-25, EORTC QLQ-30, SESCI	2 weeks before physiotherapy (baseline), 3 and 6 mth post-baseline	After 3 months, physiotherapy significantly reduced voiding and incontinence symptoms in men with prostate cancer compared to standard care. Unfortunately, the results did not persist to the 6th month of the study.
Gezginci et al. (2022), Turkey [26]	Assessment of perioperative PFMT on UI and QOL levels in men after RP	60 men after RP Con: 30 (aged 69.2 ± 5.4 yr) Exp: 30 (aged 67.6 ± 6.7 yr)	Con: standard care without physiotherapy. Exp: training on admission to the clinic—PMEs, bladder control technique, lifestyle change; PMEs for surgery and after catheter removal—continuation of PMEs for 3 months at home, telephone check once a week. Assessment: ICIQ-SF, ICIQ-MLUTS	7 days after catheter removal, 3 mth AS	Men who performed perioperative PMEs had fewer urinary problems than those in the control group. Physiotherapy also improved the quality of life of patients after RP.
Heydenreich et al. (2020), Germany [28]	Assessment of the impact of sensorimotor training with an oscillating rod compared to standard PFMT on the reduction of UI level, recovery time and quality of life in men after RP	184 men after RP Con: 91 (aged 64.3 ± 7.4 yr) Exp: 93 (aged 64.0 ± 6.5 yr)	Con: 30 min relaxation training combined with supervised PFMT. Exp: sPFFT with coordinated use of an oscillating rod (30 min). Supervised PFFT 30 min in both groups, 1 session daily for 3 wk. Assessment: 1 h + 24 h PT, HRQL, FACT-P	Before and 3 wk after therapy	Experimental training—better results and a significant reduction in UI as well as improved quality of life in men after RP. Improvement of continence in both trainings.
Van Kampen et al. (2000), Belgium [29]	Assessment of the impact of PMF reeducation on the improvement of continence in patients after RP with UI	102 men after RP Con: 52 (aged 66.58 ± 0.8 yr) Exp: 50 (aged 64.36 ± 0.81 yr) Eventually, after 1 year, 48 men from Exp. And 50 from Con. Finished the trial.	Con: education about UI after RP, false ES—false interferential current (placebo), 1 session a wk for max. year. Exp: education about PMF and urinary system, sPFMT with BF—patients who could not stretch PMF received AES, 90 contractions of PMF daily at home in 3 positions, activating PMF in daily activities. Supervised training 1× a wk for max. year. Assessment: 1 h + 24 h PT, VAS	BS, 1st, 3rd, 6th, and 12th mth after start therapy	Men who underwent PMF re-education saw faster and better results in the reduction of UI symptoms after RP than in the placebo group.
Manassero et al. (2007), Italy [32]	Assessment of the influence of early, intense and long-term PMEs on the incidence of UI in men after bladder-sparing RRP surgery	107 men after RRP Con: 53 (aged 67.9 ± 5.5 yr) Exp: 54 (aged 66.8 ± 6.3 yr) Eventually, after 1 year, 54 men from Exp. and 40 men from Con. finished the trial.	Con: no intervention. Exp: Verbal feedback to teach PMF, PMEs at home: 45 contractions (3 series of 15×)—max. 90 contractions daily, PMF activation in daily activities. Assessment: 24 h PT, VAS, PE, IPSS, QoL	1st, 3rd, 6th, and 12th mth AS	PFMT significantly influences the improvement of continence in men after RRP in comparison with control. The therapeutic effect of physiotherapy lasts for at least 12 mth and shows no side effects.
Milios et al. (2019), Australia [35]	Assessment of the effectiveness of basic PFMT compared to intense PFMT focused on the activation of slow and fast twitch fibers in men after RP/RALP	97 men after RP/RALP Con: 47 (aged 63.5 ± 6.8 yr) Exp: 50 (aged 62.2 ± 6.8 yr)	All: 2× 30 min sessions about PFMT. PFMT started 5 wk BS. To learn PMEs was used RTUS with BF. All continued PFMT for 12 wk AS. Con: 3× of PMF: contraction 10 s, relax 10 s (30× per day) in 3 positions. Exp: 6 sessions of 10 quick contractions (1 s) and 10 slow (10 s) with equal relaxation time (120× per day) in standing. Assessment: 24 h PT, bladder diary, IPSS, EPIC-CP, RTUS PFM, QoL	BS, 2nd, 6th, and 12th mth AS	Better effectiveness of therapy, reduction of UI and improvement of QoL in patients who received intensive PFMT therapy compared to patients with basic PFMT training.
Nilssen et al. (2012), Norway [37]	Effect of supervised postoperative PFMT on quality of life parameters in patients after RP	80 men after RP Gr.1: 38 (aged 60, range 48–68 yr) Gr.2: 42 (aged 62, range 49–72 yr)	Gr.1: 45 min sPFMT, 1× a wk up to 12 mth AS; at home: 3 series of PMEs in 3 positions, 10 contractions, 6–8 s each + at the end of each 3–4 quick contractions; men who could not come to the training received a DVD with PFMT (*n* = 20). Gr.2: instructions of postoperative PFMT, 3 series of 10× per day. Assessment: UCLA-PCI, SF-12	Before the therapy, 6th wk, and 3rd, 6th, and 12th mth AS	Significant reduction of postoperative UI symptoms in patients undergoing sPFMT compared to the control. No better results were obtained with HRQoL parameters.
Pedriali et al. (2015), Brazil [42]	Evaluation of the effectiveness of Pilates exercises in comparison to the traditional PFMT in the treatment of UI in men after RP	85 men after RP Gr.1: 26 (aged 66.07 ± 5.77 yr) Gr.2: 28 (aged 66.32 ± 5.48 yr) Con: 31 (aged 62.61 ± 7.26 yr)	All men were taught to work with the BF at baseline assessment. Gr.1: 10 supervised sessions of 45 min of Pilates exercises, 3 exercises and 2 Pilates exercises at home every day. Gr.2: 10 individual PFMT sessions in combination with AES, 1× a wk, 40–50 min; SUI: AES—frequency 50 Hz, 20 min; UUI—frequency 4 Hz, 20 min; MUI—both AES parameters; after each AES, 3 series of 10 strong contractions in 3 positions. Con: no intervention. Assessment: 24 h PT, 3 day bladder diary, daily pads usage, ICIQ-SF	4th wk and 4th mth AS	The Pilates method is as effective as the standard PFMT. There were no statistically significant differences in the number of pads used between men from both intervention groups in the results of the 24 h PT and ICIQ-SF. Both intervention groups achieved greater improvement over Con.
Porru et al. (2001), Italy [43]	To evaluate the effect of PFMT on UI, including urinary frequency, postmicturition dribbling, and quality of life in patients after TURP	58 men Con: 28 (aged 66.0, range 53–71 yr) Exp: 30 (aged 67.5, range 55–73 yr)	Exp: instruction, feedback about contractions, PMEs at home, 3 sessions of 15× daily. Con: no intervention. Assessment: AUA, QoL, ICS male questionnaire, uroflowmetry, digital evaluation of PMF, voiding diary	BS and 30 days AS	Men performing PFMT obtained a significantly higher degree of PMF strength, fewer UI symptoms, and better QoL than the control.
De Santana Santos et al. (2017) [46]	Analysis of the effectiveness of physiotherapy with PFMT + BF in the treatment of UI in men after RP	13 men up to 3 mth after RP Con: 6 (aged 62, range 54–74 yr) Exp: 7 (aged 65.6, range 58–70 yr)	Exp: education + instruction of PMEs at home, 1× wk for 8 wk: BF (20 min) + PMEs. Con: education + instruction of PMEs for home, PMEs in clinic. Assessment: before the start of therapy, on the 5th and 9th visits, 1 h PT		A similar reduction in UI symptoms was observed in both groups after 2 months of treatment.
Strojek et al. (2021), Poland [49]	Assessment of the effectiveness of PFMT in the treatment of SUI in men after RP	34 men Con: 15 (aged 64.2 ± 4.5 yr) Exp: 19 aged 61.4 ± 7.4 yr)	Con: no intervention. Exp: 24 individual sessions of PFMT in 3 positions, 2× a wk—2 wk AS—number of repetitions—individual; before PFMT: postural correction, mobilization of sacroiliac + sacro-lumbar joints, respiration exercises. Assessment: myostatin concentration, BDI-II, EPIC-26	At baseline and after 12th wk of therapy	PFMT significantly improves the overall quality of life of men after RP, while the lack of intervention reduces it in the ‘overall urinary problems’ and ‘sexual’ domains. PFMT also reduces the concentration of myostatin and the risk of developing depressive disorders.
Overgard et al. (2008), Norway [39]	Assessment of the impact of sPFMT on the occurrence of UI in men after RP	80 men Exp1: 38 (aged 60.0, range 48–68 yr) Exp2: 42 (aged 62.0, range 49–72 yr)	Exp1: sPFMT, 45 min a wk, at home—3 sessions of 10× in 3 positions—contraction 6–8 s + 3–4 quick contractions; instruction; men who could not come to the training received a DVD with PFMT. Exp2: instruction on postoperative training—3 sessions of 10× of PMEs. Assessment: UCLA-PCI, physiotherapeutic evaluation, 24 h PT, per rectum examination	BS, 6th wk, 3rd, 6th, and 12th mth AS	After 3 mth, no significant differences in UI were found between groups. However, after 12 mth, a significant improvement in urinary continence was observed in men who participated in sPFMT.
Tantawy et al. (2019), Egypt [50]	Effect of whole body vibration training on the occurrence of SUI in men after prostate cancer surgery	61 men Gr.1: 30 (aged 64.3 ± 5 yr) Gr.2: 31 (63.6 ± 5.8 yr)	Gr.1: PFMT + WBVT, 3× a wk for 4 wk: 1–2 session—frequency 20 Hz, peak to peak displacement of 2 mm, duration of each set of 45 s followed by 60 s rest; 3–12 session—a frequency of 40 Hz, peak-to-peak displacement of 4 mm, duration of each set of 60 s followed by 60 s rest. Gr.2: PFMT. All men received the same guidelines for PFMT: PMEs daily, in 3 positions, 10 s of contraction, 10 s of relaxation, 15×; for slow twitch fibers, the time of contraction and relaxation was increased by 1 s every wk; fast twitch fibers—quick contractions and relaxation of PMF, 20×, then 10 s of rest—initially 2 sets, finally 4 sets. Assessment: I-VAS, ICIQ-SF, 24 h PT	Before therapy, after 4 wk of treatment and after 2 mth of observation	Improvement in SUI symptoms was noted in men after both PFMT and WBVT combined therapy, as well as after PFMT alone.

AES, anal electrical stimulation; AUA, the American Urological Association Symptom Score; AS, after surgery; BF, biofeedback; BPH, benign prostatic hyperplasia; BS, before surgery; BT, brachytherapy; Con, control group; EBRT, external beam radiation; EORTC QLQ-PR25, European Organisation for Research and Treatment of Cancer Quality of Life prostate scale; EORTC QLQ-C30, European Organization for Research and Treatment of Cancer Quality of Life Questionnaire Core 30; EPIC-CP, Expanded Prostate Cancer Index Composite for Clinical Practice; ES, electrical stimulation; Exp, experimental group; FACT-P, Functional Assessment of Cancer Therapy-Prostate questionnaire; HoLEP, holmium laser enucleation of the prostate; HRQL, Health-Related Quality of Life; ICIQ-MLUTS, International Consultation on Incontinence Questionnaire for Male Lower Urinary Tract Symptoms; ICIQ-SF, International Consultation on Incontinence Questionnaire Short-Form; ICS-SF, International Continence Society male Short Form; IDQ, Incontinence Diagnosis Questionnaire; IPSS, International Prostate Symptom Score; I-VAS, Incontinence Visual Analogue Scale; MMSE, the Mini-Mental State Examination; mth, month; MUI, mixed urinary incontinence; PE, physical examination; PGI-I, Patient’s Global Impression of Improvement; PFMT, pelvic muscles floor training; PMEs, pelvic muscle exercises; PMF, pelvic muscles floor; QoL, quality of life; PT, pad test; RALP, robotic-assisted laparoscopic prostatectomy; RP, radical prostatectomy; RRP, retropubic radical prostatectomy; RTUS, real-time ultrasound; SESCI, Self-Efficacy for Symptom Control Inventory; SF-12, the Short Form-12; sPFMT, supervised pelvic muscles floor training; SUI, stress urinary incontinence; TURP, transurethral prostatectomy; UCLA-PCI, University of California, Los Angeles—Prostate Cancer Index; UI, urinary incontinence; UUI, urgency urinary incontinence; VAS, visual analog scale; WBVT, whole-body vibration training; wk, week; yr, years.

**Table 3:** PFMT training with BF in men with UI after RP.

Reference	Main Objective	Participants	Intervention	Follow-up	Outcomes
Allameh et al. (2021), Iran [17]	Assessment of the effectiveness of pre- and postoperative PFMT and BF in the treatment of UI in men after RP	57 men after RP Con: 19 (aged 70.6 ± 6.8 yr) Exp1: 19 (aged 69.0 ± 5.7 yr) Exp2: 19 (aged 68.4 ± 6.9 yr)	Con: nonfunctional probes of BF before and after RP, instruction of PFMT after RP. Exp1: 30 min of BF 2× a wk before 2 wk of RP, nonfunctional BF after RP, instruction of PFMT after RP. Exp2: nonfunctional BF before RP, 30 min of BF 2× a wk after RP, instruction of PFMT after RP. Assessment: 24 h PT	1st, 3rd, and 6th mth after catheter removal,	Compared to the lack of therapy, the use of BF before or after surgery significantly improves continence in men within 1 and 3 months after RP.
De Lira et al. (2019), Brazil [22]	Assessment of the impact of perioperative PFMT in comparison with standard care on minimizing the symptoms of UI and erectile dysfunction in men after RP	31 men after RP Con: 15 (aged 63.53 ± 7.62 yr) Exp: 16 (aged 67.3 ± 5.63 yr)	Con: no intervention. Exp: sPFMT + BF: 2 session BS, PFMT at home 3× a day BS and AS. Assessment: ICIQ-SF, IIEF-5, electromyographic recordings of the pelvic floor	Before and 3 mth AS	sPFMT and home training instructions do not minimize UI and erection problems in men after RP.
Floratos et al. (2002), the Netherlands [24]	Compare the effectiveness of EMG with verbal instructions as tools for learning PME in the early treatment of UI after RP	42 men after RP Con: 14 (aged 65.8 ± 4.3 yr) Exp: 28 (aged 63.1 ± 4.0 yr)	Con: palpation + verbal feedback, leaflet about PMEs, telephone consultations, 80–100× PMEs at home daily (4 sessions of 20–25×). Exp: 30 min, 15 series, 3× a wk EMG BF, 50–100× of PMEs daily at home. Assessment: 1 h PT, individual questionnaire, urodynamic examination at mth 6 of men with UI	1, 2, 3 and 6 mth after start therapy	Verbal feedback and BF combined with EMG are effective methods in learning PMEs in men after RP. Both methods are effective in minimizing the symptoms of UI.
Geraerts et al. (2013), Belgium [25]	Comparative evaluation of the effects of PFMT before and after ORP/RARP surgery in the treatment of UI with the effectiveness of only postoperative training	180 men after ORP/RARP Con: 89 (aged 62.04 ± 6.33 yr) Exp: 91 (aged 61.88 ± 5.90 yr) 12 months after surgery, 85 men were finally evaluated in both groups.	Con: after catheterization—1 sPFMT with EMG BF, information about PMEs, PMF activation in everyday activities. Exp: 3 wk BS—sPFMT EMG BF training, 3× 30 min, 1× a wk, PMEs at home 60 contractions, activation PMF in everyday activities; PFMT 4 days AS. Assessment: 1 h + 24 h PT, VAS, IPSS, KHQ	BS, 1st, 3rd, 6th, and 12th mth AS	Pre- and postoperative as well as exclusively postoperative PFMT show a similar therapeutic effect in the treatment of UI in men after ORP or RARP.
Moore et al. (2008), Canada [36]	Assessment of the effectiveness of PFMT in comparison to telemedicine with a urology nurse in men after RP	Con: 77 Exp: 89	Con: contact with nurse, verbal, and written instruction about PFMT: 5–10 s contraction, 10–20 s relaxation, 12–20 repetitions, 3× a day at home. Exp: verbal and written instructions about PFMT, BF training: 30 min, 1× a wk—strength: 5–10 s contraction, 10–20 s rest, 12–20×; endurance: 50–60% of max strength, 20–60 s contraction and relaxation, 6–8×; speed: 5–10 contraction during 10 s, 20 s rest period; control: contractions in 3 stages, 15 s of slow release, 15 s rest, 6–10×; penile lift exercises + 3× a day at home on nontreatment days. Assessment: 24 h PT, IPSS, IIQ-7	At baseline, 4th, 8th, 12th, 16th, 26th wk and 1 yr	At individual stages of the assessment, both groups showed a similar improvement of continence in men.
Oh et al. (2020), Korea [38]	Evaluation of the effectiveness of the innovative BF device—Anykegel in PFMT in men with UI after RARP	82 men Con: 42 (aged 65.9 ± 6.8 yr) Exp: 40 (67.5 ± 6.9 yr)	Con: verbal and written instruction—instructions were given in three different way—4× a day, 10 min of exercises, min. 10 s of tension duration and max. tension intensity. Exp: verbal and written instruction + BF Any kegel, 4× a day, 10 min of exercises session, 10 s of tension. Assessment: physical examination, 24 h PT, IPSS, IIEF-5	BS, 1st, 2nd, and 3rd mth after catheter removal	BF has a significant impact on the treatment of UI in men after RARP, especially in the early postoperative period.
Perez et al. (2018), Brazil [41]	Assessment of BF as a preventive measure against UI and erectile dysfunction in men after RP	52 men after RP Con: 32 (aged 66.3 ± 5.8 yr) Exp: 20 (aged 64.0 ± 4.6 yr)	Con: no intervention. Exp: BF BS; the therapy started with pressure taring—3 max. PMF contractions, followed by 7 min fast and 6 min slow; 10 sessions. Assessment: KHQ, IIEF-5	Before therapy and AS	Men exercising PFMT with BF before the surgery suffered significantly less for UI and erectile dysfunction than the control.
Rajkowska-Labon et al. (2014), Poland [44]	Evaluation of the effectiveness of physiotherapeutic methods in comparison to the lack of therapy in the treatment of UI in men after RP	81 men after RP Con: 32 (aged 68.3 ± 6.49 yr) Exp1: 23 (aged 66.9 ± 7.07 yr) Exp2: 26 (aged 68.8 ± 6.59 yr)	Con: no intervention. Exp1: PFMT + BF, 1× a wk, 20–30 min; PFMT + SSS, 1× a wk, 30 min; PFMT in 3 positions at home, 3× a day, 15–20 min. Exp2: PFMT + SSS, 2× wk, 30 min; PFMT at home (the same as Exp1.). Assessment: 1 h + 24 h PT, sEMG, patients’ self-reported subjective assessment	Exp 1. + 2: at baseline and end of therapy (max. 1 yr), Con: at baseline and 1 yr AS	Physiotherapeutic treatment significantly reduces the symptoms of RUI after RP compared to no treatment. However, treatment with PFMT + SSS resulted in no UI problems among 92.3% of men, while PFMT + BF only of 39.1%.
Serdà et al. (2014), Spain [47]	Design and implementation of the PFMT program to improve UI	66 men Con: 33 (aged 71.78 ± 6.82 yr) Exp: 33 (aged 71.09 ± 8.1 yr)	Con: no intervention. Exp: global postural re-education, PFMT + BF, exercises to radiate muscular strength—24 wk—16 wk with specialists + 8 wk of autonomous training, 2× for wk, time: 60 min. Assessment: 20-min nappy test, VAS-UI, FACT-P, the waist perimeter, muscular resistance (8RM)	At baseline and at the end of therapy (24 wk)	The rehabilitation program combined with PFMT significantly improves the symptoms of UI in patients with prostate cancer. Improvement of UI problems correlates with an improvement in the quality of life of patients.
Tienforti et al. (2012), Italy [51]	Evaluation of the effectiveness of preoperative BF + low-intensity program of postoperative perineal physiokinesitherapy in reducing the frequency, duration and severity of UI in men after RP	32 men after RRP Con: 16 (aged 67, range 60–74 yr) Exp: 16 (aged 64, range 52–74 yr)	Exp: PMF education, supervised BF training the BS and after catheter removal (BF: 20 min, 1× a month), PFMT instructions, home exercises: 3× 10 min a day, 5 s contraction, 5 s relaxation. Con: standard care, after catheterization: instructions of PMEs at home—3× a day for 10 min until achieved continence. Assessment: ICIQ-Overactive Bladder, UCLA-PCI, IPSS-QOL, ICIQ-UI	Exp. assessed at each mth visit, Con. after 1, 3, and 6 mth after catheter removal	Significant improvement after 3 and 6 mth in the number of pads used and the number of UI episodes in patients in the intervention group compared to the control.
Ribeiro et al. (2010), Brazil [45]	Assessment of the effectiveness of PFMT with BF method in improving UI in men within 12 mth after RP	73 men after RP Con: 37 (aged 65.6 ± 8.0 yr) Exp: 36 (aged 62.2 ± 6.3 yr) 54 patients were included in the final evaluation Con: 28 Exp: 26	Exp: From the 15th day AS BF-PFMT treatment, 1× a wk, for a max. 12 wk or until the symptoms of UI stop. 30 min session, BF-PFMT with electromyographic machine. Instructions for daily home training in 3 positions. Con: instruction from a urologist, no recommendations. Assessment: number of pads/day, 24 h PT, ICSI, ICST, IIQ-7, QOL, the Oxford scale.	BS, 1st, 3rd, 6th, and 12 mth AS	The implementation of early BF-PFMT in men after RP significantly improves continence, reduces the frequency of episodes, and improves the strength of PFM 12 mth after surgery compared to the control.
Zhang et al. (2015), USA [54]	Assessment of the effectiveness of combining PFMT with a symptom self-management in reducing UI symptoms in patients with prostate cancer	244 men Con: 82 (aged 64.9 ± 8.2 yr) Exp1: 81 (aged 66.8 ± 7.2 yr) Exp2: 81 (aged 64.3 ± 7.3 yr)	Con: usual care, without any intervention. Exp1: PFMT + BF + support group + PST: 3–5 participants, time: 60–75 min; 6 biweekly sessions for 3 mth. Exp2: PFMT + BF + individual telephone contact with a therapist, time + PST: 45 min; 6 biweekly sessions for 3 mth. Assessment: ICSmaleSF, SPMSQ	At baseline, 3 mth after intervention and at 6 mth	Both intervention groups showed a lower frequency of daily leakage of urine after 3 mth (but not 6 mth) than in the control. However, after 6 mth, they reported fewer UI problems than the men in the control group.

AS, after surgery; BF, biofeedback; BS, before surgery; Con, control group; EMG BF, electromyographic biofeedback; Exp, experimental group; FACT-P, Functional Assessment Cancer Therapy Scale Prostate; ICIQ-UI, International Consultation on Incontinence Modular Questionnaire—Urinary Incontinence; ICIQ-SF, International Consultation on Incontinence Questionnaire, Short-Form; ICSI, Incontinence Symptoms of the International Continence Society Male Short Form questionnaire; ICSmaleSF, International Continence Society Male Short Form; ICST, total score of the International Continence Society Male Short Form questionnaire; IIQ-7, Incontinence Impact Questionnaire-7; IIEF-5, the International Index of Erectile Function; IPSS, International Prostate Symptom Score; IPSS-QOL, International Prostate Symptom Score—Quality of Life; KHQ, King’s Health Questionnaire; mth, month; ORP, open radical prostatectomy; PFMT, pelvic muscles floor training; PMEs, pelvic muscle exercises; PMF, pelvic muscles floor; PST, problem solving therapy; PT, pad test; QOL, quality of life; RARP, robot-assisted laparoscopic radical prostatectomy; RP, radical prostatectomy; RRP, retropubic radical prostatectomy; sPFMT, supervised pelvic muscles floor training; SPMSQ, Short Portable Mental Status Questionnaire; SSS, spinal segmental stabilization; UCLA-PCI, University of California, Los Angeles—Prostate Cancer Index; UI, urinary incontinence; VAS, visual analog scale; yr, years.

**Table 4:** Electrostimulation in men with UI after RP.

Reference	Main Objective	Participants	Intervention	Follow up	Outcomes
Ahmed et al. (2012), Egypt [16]	Assessment of the influence of PFMT, ES and BF on the occurrence of UI in men after RP	80 men after RP Exp1: 26 (aged 57.2 ± 3.25 yr) Exp2: 26 (aged 58.8 ± 5.4 yr) Exp3.: 28 (aged 56.3 ± 6.8 yr)	Exp1: instructions + leaflet with PMEs—3 series of 15–20× a day. Exp2: 15 min, 2× a wk (12 wk); frequency: 50 Hz; pulse width: 300 μs; intensity: maximum tolerated. Exp3: 15 min BF + 15 min ES, 2× a wk (12 wk), 3× of 10 quick contractions; 3 contractions for 5, 7, or 10 s; 10 contractions on prolonged exhalation. Assessment: 24 h PT, IIQ-7, urodynamic test only in men with UI after 6 mth	wk after catheter removal, at 6 and 12 wk during intervention, and 24 wk after catheterization	Improvement in continence was noted in all study groups. The greatest effect was obtained by men undergoing combined therapy—BF + ES, both for the duration and degree of UI and QoL.
Gomes et al. (2018), Brazil [27]	Assessment of the impact of Pilates exercise compared to the conventional PFMT protocol on pelvic floor muscle strength in patients with UI after RP	104 men after RP Gr.1: 34 (aged 66.62 ± 5.66 yr) Gr.2: 35 (aged 65.83 ± 5.64 yr) Con: 35 (aged 63.11 ± 7.19 yr)	Gr.1: 10 supervised Pilates training—1× a wk, 45 min; instructions for daily home exercises. Gr.2: SUI: AES frequency 50 Hz, 20 min; UUI: frequency 4 Hz, 20 min; MUI: both of the above electrical parameters. All performed after AES PFMT—3× of 10 contractions—10 s, PFMT, once a wk, 45 min; instructions for daily home exercises. Con: no intervention. Assessment: 24 h PT, ICIQ-SF, a manometric perineometry, voiding diary	Before and 4 mth AS	The improvement in PFMT parameters was greater in the actively treated groups compared to the control group. Traditional PFMT combined with AES and the Pilates method have a similar effectiveness in minimizing UI symptoms in men after RP.
Laurienzo et al. (2018), Brazil [30]	Assessment of the effect of ES and PFMT on muscle strength, erection, and UI in men with prostate cancer treated with RP	123 men after RP Con: 40 (aged 57.3 ± 6.5 yr) Gr.1: 41 (aged 58 ± 5.7 yr) Gr.2: 42 (aged 58.5 ± 5.4 yr)	Con: information on postoperative management. Gr.1: 3 types of PMEs at home, 2–3× daily for 6 mth. Gr.2: PMEs (identical to Gr.1), AES, 2× a wk, for 7 wk, frequency 35 Hz, pulse width 1 ms, rise time 2 s, stimulus duration 6 s, fall time 2 s, standing time 12 s, intensity adapted to the patient. Assessment: 1 h PT, ICIQ-SF, IIEF-5, IPSS, perineometer	BS, 1st, 3rd, and 6th mth AS	After 6 months of the study, an improvement in the strength of PMF, a reduction in UI symptoms and erectile dysfunction in men from each group was shown. Nevertheless, no statistical differences were found between the groups.
Laurienzo et al. (2013), Brazil [31]	Evaluation of the effectiveness of the use in the treatment of UI of preoperative ES in men after RRP	49 men after RRP Con: 15 (aged 64.0 ± 8 yr) Gr.1: 17 (62.0 ± 7 yr) Gr.2: 17 (60.0 ± 8 yr)	Con: instruction about PMEs. Gr.1: Kegel exercises. Gr.2: BS: PMEs—5 s contraction in 3 positions for 10× + 10× ES: tonic fibers—frequency 20 Hz, pulse width 700 μs, rise time 2 s, descent time 2 s, working time 6 s, rest time 6 s, time 10 min; phase fibers—frequency 65 Hz, pulse width 150 μs, rise time 2 s, descent time 2 s, working time 6 s, rest time 18 s, time 5 min. Assessment: 1 h PT, ICIQ-SF	1st, 3rd, and 6th mth AS	There were no significant differences between men in the level of UI and QoL.
Mariotti et al. (2009), Italy [33]	Analysis of the benefits of the early FES and BF therapy in terms of recovery time and improvement in continence in men after RP	60 men after RP Con: 30 (aged 61.43 ± 3.60 yr) Exp: 30 (aged 61.86 ± 3.26 yr)	Con: PMF instruction, written examples of Kegel exercises. Exp: 2 sessions for 6 wk: BF—15 min, FES—frequency 30 Hz for 10 min, then 50 Hz for 10 min—all 20 min, pulse duration 300 μs, max. output 24 mA, intensity adjusted to the patient. Assessment: 24 h PT, ICS-male questionnaire	Before therapy; at 2nd and 4th wk; and 2nd, 3rd, 4th, 5th, and 6th mth after start of therapy	Early, non-invasive physical treatment with the BF and FES of the pelvic floor has a significant positive effect on the improvement of UI in men after RP between the 4th wk and the 6th mth of follow-up. One year AS 58 out of 60 men did not suffer from UI.
Mariotti et al. (2015), Italy [34]	Assessment of the effectiveness of FES + BF therapy in terms of recovery time and rate of continence in men with UI after RP	120 men RP Exp1: 60 (aged 59.61 ± 4.03 yr) Exp2: 60 (aged 59.28 ± 4.19 yr)	In both groups: FES + BF: 2× a wk for 6 wk, BF—15 min + verbal guidance, exercises in 3 positions, FES—20 min—pulsed at 30 Hz (first 10 min) + 50 Hz (second 10 min), square waves—300-μs pulse duration + max. output—24 mA, intensity adjusted to the patient. Exp1: started 14 days after catheter removal. Exp2: AS—verbal and written instruction of PFMT; FES + BF—12 mth AS. Assessment: 24 h PT, ICS-male questionnaire	Time 0—before therapy and 14 days after catheter removal—Exp1. and 12 mth AS—Exp2.; at 2 and 4 wk and 2, 3, 4, 5, and 6 mth after start of treatment	FES + BF therapy significantly reduces the symptoms of UI in men after RP, regardless of the time of its initiation.
Pané-Alemany et al. (2021), Spain [40]	Assessment of the effectiveness of transcutaneous perineal electrostimulation and intracavitary electrostimulation in the treatment of UI and the impact on QOL in men after RP	70 men after RP Con: 35 (aged 62.7 ± 10.2 yr) Exp: 35 (aged 62.9 ± 8.8 yr)	Con: transcutaneous perineal electrostimulation, PFMT. Exp: anal electrostimulation, PFMT. Parameters in both groups: 15 min—10 min, frequency 30 Hz, pulse width 0.25 ms, intensity 10–30 mA, no on-off cycles; 5 min—frequency 50 Hz, pulse width 0.25 ms, intensity 1–50 mA, individually time of on-off cycles; PFMT with physiotherapist and at home: 20 contractions (10× 8–10 s, 10× 3 s), 3× daily for 10 wk. Assessment: physical examination with OXFROD scale, 24 h PT, ICIQ-SF, SF-12, I-QoL test	Baseline, at 6 and 10 session	Regardless of the type of electrostimulation, improvement in continence and quality of life was noted in both groups.
Soto-González et al. (2020), Spain [48]	Analysis of the effectiveness of 3-month ES and BF therapy in the treatment of UI in patients after RP	47 men after RP Con: 22 Exp: 25	All -instruction of PMF exercises at home. Exp: ES and BF, 3× a wk for 3 months, ES: 15 min, 300 ns pulse duration, maximum intensity 24 mA; BF: 30 min. Con: no intervention. Assessment: 1 h and 24 h PT, urinary diary, ICIQ-SF	Before therapy, after 1st, 2nd, 3rd, and 6th mth of therapy	After 3 months of treatment was observed a positive effect of the combined therapy (ES and BF) on the occurrence of UI in men after RP. Moreover, it also leads to an improvement in the QoL.
Yamanishi et al. (2010), Japan [52]	Evaluation of the effectiveness of ES therapy combined with PFMT in the treatment of UI in patients after RP	56 men after RP Con: 30 (aged 68.0 ± 5.6 yr) Exp: 26 (aged 65.4 ± 5.6 yr)	All performed preoperative PFMT and continued it. Exp: AES: 15 min, 2× a day, frequency 50 Hz, pulse duration 300 μs, max. output 70 mA (5 s on, 5 s off). Con: sham AES: 15 min, 2× a day, frequency 50 Hz, pulse duration 300 μs, max. output 3 mA (2 s on, 13 s off). Assessment: 3-day PT, ICIQ-SF, KHQ	wk after catheterization, 1, 3, 6, and 12 mth from the start of therapy	The continence rate was significantly higher in men with active AES than with sham AES after 1, 3, and 6 mth of treatment. However, similar differences were not shown at 12 mth, while at 6 mth the difference was small.
Yokoyama et al. (2004), Japan [53]	Assessment of the effectiveness of ExMI and FES in the treatment of UI in men after RRP	36 men after RRP FES: 12 (67.2 ± 6.7 yr) ExMI: 12 (68.2 ± 4.9 yr) Con: 12 (66.2 ± 7.6 yr)	FES: 15 min, 2× a day for a mth, pulses of 20 Hz square waves at a 300 s pulse duration and a max. output current of 24 mA. ExMI: 20 min, 2× a wk for 2 months, the frequency 10 Hz, intermittently for 1 min, followed by a rest period of min, a second treatment at 50 Hz intermittently for 10 min. Con: PFMT learning (rectal examination), instructions for home exercises. Assessment: bladder diaries, 24 h PT, quality of life survey	1, 2 and 4 wk and 2, 3, 4, 5 and 6 mth after catheter removal	FES and ExMI therapy enables faster UI improvement in men after RRP than at home PFMT.

AES, anal electrical stimulation; AS, after surgery; BF, biofeedback; BS, before surgery; Con, control group; ES, electrical stimulation; ExMI, extracorporeal magnetic innervation; Exp, experimental group; FES, pelvic floor electrical stimulation; ICIQ-SF, International Consultation on Incontinence Questionnaire, Short-Form; ICS-male questionnaire, The International Continence Society—male questionnaire; IIEF-5, the International Index of Erectile Function; IIQ-7, Incontinence Impact Questionnaire -7; IPSS, International Prostate Symptom Score; KHQ, King’s Health Questionnaire; mth, month; MUI, mixed urinary incontinence; PFMT, pelvic muscles floor training; PMEs, pelvic muscle exercises; PMF, pelvic muscles floor; PT, pad test; QoL, quality of life; RP, radical prostatectomy; RRP, retropubic radical prostatectomy; SF-12, Short Form Health Survey; sPFMT, supervised pelvic muscles floor training; SUI, stress urinary incontinence; UI, urinary incontinence; UUI, urgency urinary incontinence; wk, week; yr, years.

**Figure A1:**
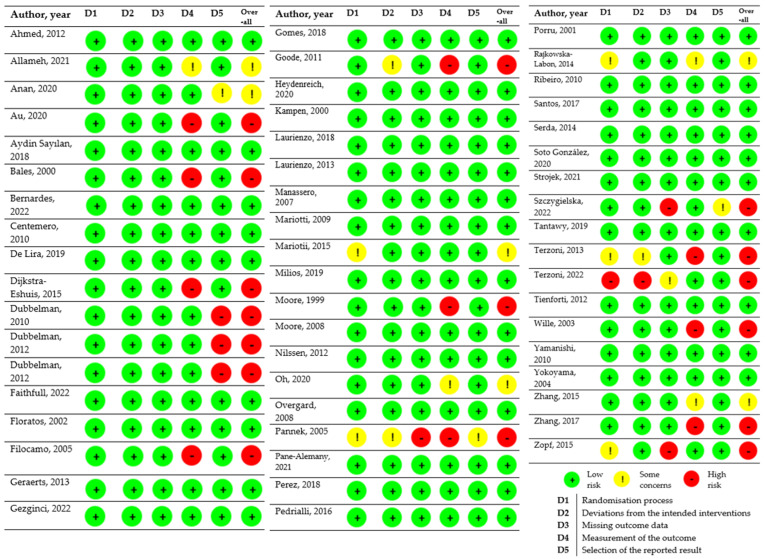
RoB analysis (*n* = 55). Low risk studies: [16,19,20,21,22,23,24,25,26,27,28,29,30,31,32,33,35,36,37,39,40,41,42,43,45,46,47,48,49,50,51,52,53], some concerns studies: [17,18,34,38,44,54], high risk studies [62,72,73,74,75,76,77,78,79,80,81,82,83,84,85,86].

**Figure A2:**
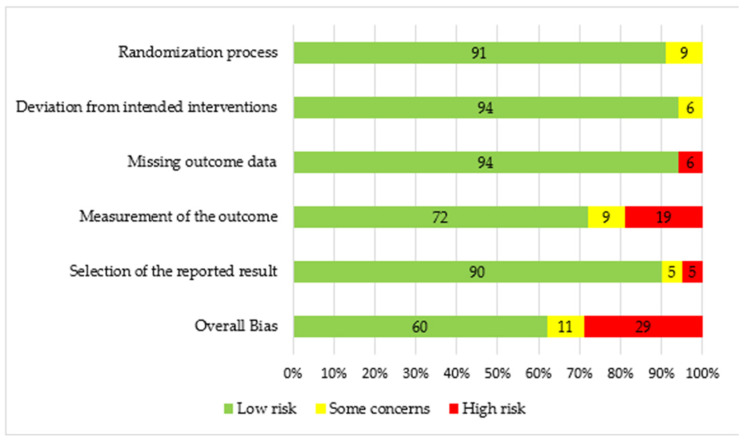
Summary plot of the overall risk of bias (*n* = 55).

## Data Availability

The data underlying this article will be shared on reasonable request to the corresponding author.

## Appendix A

Based on the RoB-2 risk of bias assessment tool, 33 studies were assessed as ‘low risk’, 5 as ‘some concern’, and 16 as ‘high risk’. Figure A1 presents risk of bias in every domain and overall. Figure A2 shows total percentage plot of the overall risk of bias.
